# Solving the RNA polymerase I structural puzzle

**DOI:** 10.1107/S1399004714015788

**Published:** 2014-09-27

**Authors:** María Moreno-Morcillo, Nicholas M. I. Taylor, Tim Gruene, Pierre Legrand, Umar J. Rashid, Federico M. Ruiz, Ulrich Steuerwald, Christoph W. Müller, Carlos Fernández-Tornero

**Affiliations:** a Structural and Computational Biology Unit, European Molecular Biology Laboratory, Meyerhofstrasse 1, 69117 Heidelberg, Germany; b Centro de Investigaciones Biológicas, Consejo Superior de Investigaciones Científicas, Ramiro de Maeztu 9, 28040 Madrid, Spain; c Department of Structural Chemistry, Georg-August-University, Tammannstrasse 4, 37077 Göttingen, Germany; d SOLEIL Synchrotron, L’Orme de Merisiers, Saint Aubin, Gif-sur-Yvette, France

**Keywords:** low-resolution structure determination, multi-subunit complexes, transcription, RNA polymerase I

## Abstract

Knowing the structure of multi-subunit complexes is critical to understand basic cellular functions. However, when crystals of these complexes can be obtained they rarely diffract beyond 3 Å resolution, which complicates X-ray structure determination and refinement. The crystal structure of RNA polymerase I, an essential cellular machine that synthesizes the precursor of ribosomal RNA in the nucleolus of eukaryotic cells, has recently been solved. Here, the crucial steps that were undertaken to build the atomic model of this multi-subunit enzyme are reported, emphasizing how simple crystallographic experiments can be used to extract relevant biological information. In particular, this report discusses the combination of poor molecular replacement and experimental phases, the application of multi-crystal averaging and the use of anomalous scatterers as sequence markers to guide tracing and to locate the active site. The methods outlined here will likely serve as a reference for future structural determination of large complexes at low resolution.

## Abstract 2

Details of the RNA polymerase I crystal structure determination provide a framework for solution of the structures of other multi-subunit complexes. Simple crystallographic experiments are described to extract relevant biological information such as the location of the enzyme active site.

## 1. Introduction   

The vast majority of cellular processes are not carried out by individual proteins; instead, these macromolecules assemble to act in a coordinated manner (Alberts, 1998 ▶). However, the large size, miscellaneous composition and conformational dynamism that characterize macromolecular complexes impose limitations on their structural analysis (Dyda, 2010 ▶). To achieve this goal, X-ray crystallography and electron microscopy (EM) have taken converging paths. While EM tools have been developed to push the resolution up to the quasi-atomic level (Amunts *et al.*, 2014 ▶; Wong *et al.*, 2014 ▶), X-ray crystallographic methods aim to squeeze out information from low-resolution data (Pomeranz Krummel *et al.*, 2009 ▶). The latter is owing to the fact that crystals of multi-subunit complexes usually diffract to limited resolution and in such cases standard quasi-automated procedures often fail, making it necessary to push the available methodologies to the limit.

RNA synthesis in the nucleus is performed by three different RNA polymerases (Pols). Pol I transcribes ribosomal DNA, Pol II produces all messenger RNAs and Pol III synthesizes transfer and other small nontranslated RNAs. Pol I-mediated transcription is critical to regulate cell growth (Grummt, 2003 ▶). Accordingly, Pol I is the most active eukaryotic RNA polymerase, contributing up to 60% of the total transcriptional activity (Warner, 1999 ▶). Moreover, alterations in cell proliferation correlate with changes in ribosomal RNA synthesis and thus misregulation of mammalian Pol I is associated with different types of cancer (Moss *et al.*, 2007 ▶).

Pol I, Pol II and Pol III are macromolecular complexes with overall masses of above 500 kDa consisting of 14, 12 and 17 subunits, respectively (Table 1 ▶). Five of these polypeptides, accounting for 10–15% of the total mass, are common to all three enzymes. In addition, Pol I and Pol III share the AC40/AC19 heterodimer, which is homologous to Rpb3/Rpb11 in Pol II. Among the seven Pol I-specific subunits, three show significant homology to their counterparts in the other cellular RNA polymerases: A190 and A135 interact with each other to form the DNA-binding cleft and the active site, while A12.2 reaches the cleft to assist in RNA cleavage during backtracking. Two less conserved specific subunits form the stalk heterodimer A43/A14 that is involved in Pol I dimerization. Finally, the A49/A34.5 heterodimer has no counterpart in Pol II but shows homology to specific regions in the transcription factors TFIIF and TFIIE (Geiger *et al.*, 2010 ▶).

While different crystal structures of Pol II have been determined in the past dozen years that have nearly allowed a dynamic view of the transcription process (Cheung & Cramer, 2012 ▶), atomic structural information on the complete Pol I enzyme has been lacking. We were able to solve the structure of this essential macromolecular complex from crystals belonging to three different forms in space group *C*2, all of which diffracted beyond 3.5 Å resolution (Fernández-Tornero *et al.*, 2013 ▶). The corresponding atomic models were fully refined and deposited in the PDB as entries 4c3h, 4c3i and 4c3j, while a very similar structure has also been determined (Engel *et al.*, 2013 ▶). Several nonstandard approaches were undertaken to achieve our results. While a partial molecular-replacement solution could be obtained, experimental phasing was necessary to yield proper electron-density maps. Moreover, we turned the appearance of various crystal forms to our advantage, thus improving electron densities through multi-crystal averaging. Using partial labelling of yeast cells with selenomethionine (SeMet), we obtained sequence markers to assist in model building in different areas of the structure.

In the present report, we describe the various difficulties encountered during the Pol I structure-determination process and the steps that were taken to overcome them. We also show that relevant biological information such as Pol I dimerization, widening of the DNA-binding cleft and the location of structural and functional ions can be gained at limited resolution. We trust that this report will serve the crystallographic community in providing a framework for similarly ambitious projects on large macromolecular complexes.

## 2. Experimental procedures   

### 2.1. Yeast strains and fermentation   

*Saccharomyces cerevisiae* strain SC1613, encoding a tandem affinity-purification (TAP) tag at the C-terminus of subunit AC40, was provided by Cellzome AG (Heidelberg, Germany). Yeast cells were grown on a fresh YPD plate and then transferred to a 50 ml flask of YPD with 0.05% adenine sulfate (YPDA) and incubated for 24 h at 30°C and 300 rev min^−1^. This pre-inoculum was seeded into 500 ml of the same medium, which after overnight incubation in identical conditions was used to inoculate 30 l YPDA. Cells were grown in a BIOSTAT Cplus fermentor (Sartorius) for 16 h at 30°C and 180 rev min^−1^ to an OD_600_ of 5–6, harvested by centrifugation and stored at −80°C until use.

For optimized selenomethionine (SeMet) labelling, cells were pre-adapted to a medium containing 80 mg l^−1^ SeMet. Cells were first grown on a YPD plate and inoculated into 50 ml modified synthetic complete medium (MSCM) composed of 13.4 g l^−1^ yeast nitrogen base without amino acids (Difco), 30 g l^−1^ d-glucose (Merck) and 5.28 g l^−1^ amino-acid mix (Formedium) complemented with 100 mg l^−1^ methionine. Cells were incubated for 24 h at 30°C and 300 rev min^−1^ and were then transferred to 200 ml MSCM with 80 mg l^−1^ SeMet. After 4 d growth in identical conditions, the cells were diluted with fresh MSCM with 80 mg l^−1^ SeMet, grown for a further 24 h and stocked in glycerol at −80°C. Fermentation was started from 50 ml pre-cultures of pre-adapted cells in MSCM with 40 mg l^−1^ SeMet. After overnight growth at 30°C and 300 rev min^−1^, the cells reached an OD_600_ of 4–6 and were used to inoculate 300 ml MSCM with 40 mg l^−1^ SeMet, followed by incubation at 30°C and 200 rev min^−1^ for 6–8 h. This was used to seed 32 l of the same medium that was grown in a fermentor under controlled oxygen and pH conditions. Cells were harvested at an OD_600_ of 5.5 and stored at −80°C until use.

### 2.2. Protein purification   

For purification, 1 kg of cells was suspended in buffer *A* (250 m*M* Tris–HCl pH 8, 40% glycerol, 250 m*M* ammonium sulfate, 1 m*M* EDTA, 10 m*M* MgCl_2_, 10 µ*M* ZnCl_2_, 12 m*M* β-mercaptoethanol) supplemented with protease-inhibitor cocktail (cOmplete EDTA-free, Roche) and lysed at 4°C with glass beads in a BeadBeater (BioSpec). The soluble fraction obtained after centrifugation (1 h at 14 000 rev min^−1^ in a Beckmann JA14 rotor) was loaded onto Heparin Sepharose (GE Healthcare) equilibrated in buffer *A*. The column was washed with buffer *B* (50 m*M* Tris–HCl pH 8, 250 m*M* ammonium sulfate, 0.5 m*M* EDTA, 1 m*M* MgCl_2_, 10 µ*M* ZnCl_2_, 1 m*M* β-mercaptoethanol, 0.5 m*M* PMSF) and the complex was eluted from the resin with buffer B* (buffer *B* with 1 *M* ammonium sulfate). The sample was diluted to 500 m*M* ammonium sulfate and incubated with 10 ml pre-equilibrated IgG Sepharose (GE Healthcare) for 6 h. After washing with ten column volumes of buffer *C* (50 m*M* Tris–HCl pH 8, 20% glycerol, 225 m*M* ammonium sulfate, 0.5 m*M* EDTA, 1 m*M* MgCl_2_, 10 µ*M* ZnCl_2_, 2 m*M* β-mercaptoethanol, 1 mg ml^−1^ Pefabloc), the IgG beads were mixed with *Tobacco etch virus* (TEV) protease and incubated overnight at 4°C in the same buffer. The supernatant was recovered and the resin was further washed with ten column volumes of buffer C* (buffer *C* without glycerol and with only 60 m*M* ammonium sulfate). The sample was subsequently purified by ion exchange on a Mono Q column (GE Healthcare); elution was performed using a gradient from 60 m*M* to 1 *M* ammonium sulfate in buffer *D* (40 m*M* Tris–HCl pH 8, 0.5 m*M* EDTA, 1 m*M* MgCl_2_, 10 µ*M* ZnCl_2_, 1 mg ml^−1^ Pefabloc, 10 m*M* DTT). Pol I and Pol III eluted at ∼250 and ∼350 m*M* ammonium sulfate, respectively. The sample was concentrated to 6.5–7 mg ml^−1^ before crystallization.

### 2.3. Crystal growth and derivatization   

Initially, commercial sparse-matrix screens from Qiagen, Hampton Research and Jena Bioscience were used in 96-well plates with sitting drops made by mixing 0.1 µl screening solution with 0.1 µl protein solution with a Mosquito robot (TTP Labtech) followed by incubation at 18°C. Grid-screen optimization was performed in the same conditions. The best results were reproduced in 24-well sitting drops by mixing 1 µl protein solution and crystallization solution and incubating at 18°C, yielding crystals after 4–7 d. For crystal form C2-93, the reservoir consisted of 16–30% ethylene glycol (EG), 100 m*M* MES pH 6.3–6.9, while crystals of forms C2-90 and C2-100 grew in 1–12.5% MPD, 100 m*M* Tris–HCl pH 6.3–6.9. Cryoprotection was achieved by either a stepwise increase of the EG concentration to 30% or by soaking crystals in a solution with 22.5% MPD. All crystals were cooled in cryoloops in a nitrogen stream at 100 K. For initial phasing, a native C2-93 crystal was soaked for 1 h in 30% EG containing 2 m*M* Ta_6_Br_12_ (Jena Bioscience). For advanced phasing, a native C2-90 crystal was soaked for 2 min in 22.5% MPD with 100 m*M* Yb-HPDO3A from NatX-Ray/Jena Bioscience followed by back-soaking in the cryoprotectant solution for 1 min. For active-site location, a native C2-93 crystal was soaked for 1 h in 30% EG containing 1 m*M* phenyl lead followed by back-soaking in the cryoprotectant solution.

### 2.4. Data collection and structure determination   

Diffraction data were collected at the synchrotrons listed in Tables 2 ▶, 3 ▶ and 4 ▶ and were processed using *XDS* (Kabsch, 2010 ▶). For data processing, the crystal-to-detector distance was fixed during integration to yield more reliable unit-cell parameters including error estimates. Molecular replacement was performed with *Phaser* (McCoy, 2007 ▶) using standard settings. Crystal structures of Pol II were used for initial tests: ten subunits with open (PDB entry 1i3q) and closed (PDB entry 1i50) clamps and 12 subunits (PDB entry 1wcm) (Cramer *et al.*, 2001 ▶; Kettenberger *et al.*, 2004 ▶). For sequential molecular replacement, the Pol II structure was divided into the following modules (for domain boundaries, see Cramer *et al.*, 2001 ▶). ‘Region 1’ comprises Rpb2 (except for the lobe and clamp domains), the Rpb3/Rpb11 heterodimer, Rpb10 and Rpb12. ‘Region 2’ includes the Rpb1 cleft domain, Rpb5, Rpb6 and the Rpb7/Rpb4 stalk. ‘Region 3’ comprises the active site, pore 1, funnel and dock domains of Rpb1 and subunit Rpb8. The ‘Clamp core’ includes the corresponding domains of Rpb1 and Rpb2, while the ‘Lobe’ contains the Rpb2 lobe domain. Poorly conserved domains (jaw, foot and clamp head) were excluded from the models.

Experimental phasing was performed with *SHARP* (Bricogne *et al.*, 2003 ▶) with heavy atom positions obtained by cross-difference Fourier analysis from model phases using the program *FFT* from the *CCP*4 suite (Winn *et al.*, 2011 ▶). For initial SIRAS phasing of C2-93, the native 1 data set and a Ta_6_Br_12_ (Jena Bioscience) derivative were combined. For advanced MIRAS phasing of C2-93, we used the native 2 data set, the Ta_6_Br_12_ derivative, a Yb-HPDO3A derivative and data collected at the Zn edge. For MAD phasing of C2-90, a Yb-HPDO3A derivative was used. In all cases, *f*′ and *f*′′ for peak and inflection wavelengths were determined by energy scans, while default values were used for remote wavelengths. *f*′ and *f*′′ were fixed during refinement except in the initial SIRAS phasing, where only *f*′ was fixed. The heavy atom positions, *B* factors and occupancies were refined in all cases, while the model phases were only used for parameter refinement in MIRAS. After each phasing protocol, phases were improved by solvent flattening with *SOLOMON* (Abrahams & Leslie, 1996 ▶) and *DM* (Cowtan & Main, 1996 ▶) using a mask calculated from the model as implemented in *SHARP*. Multi-crystal averaging was performed using *RESOLVE* (Terwilliger & Berendzen, 1999 ▶) as implemented in *PHENIX* (Adams *et al.*, 2010 ▶), including *B*-factor sharpening of the data with *B* factors ranging from 34 to 100 Å^2^. In this procedure, the model was divided into 28 groups as follows (for domain boundaries, see Fernández-Tornero *et al.*, 2013 ▶). A190 was split into clamp, active site + dock + pore 1, funnel, cleft + foot and jaw, A135 was split into protrusion + fork, lobe + external, hybrid binding + wall and anchor + stalk binding + clamp, AC40 was divided into dimer + 4Fe4S-like and domain 2, A43 was divided into N-terminal, tip, OB domain and C-terminal tail, A12 was split into N-terminal Zn ribbon, linker and C-terminal Zn ribbon, and Rpb12 was split into Zn ribbon and C-terminal tail; the remaining subunits were treated independently.

### 2.5. Model building and refinement   

Model building was performed with *Coot* (Emsley *et al.*, 2010 ▶) using secondary-structure restraints and strong geometry weights during real-space refinement. Refinements in *PHENIX* (Adams *et al.*, 2010 ▶) and *REFMAC*5 (Murshudov *et al.*, 2011 ▶) were run as a grid screen to evaluate the effects of different variables. The results of parameter changes were evaluated based on the r.m.s. deviations of bonds and angles, figure of merit, log-likelihood gain, *R*_work_ and *R*_free_ values and model geometry, and only the best trial was taken for the next building round. External restraints for *REFMAC*5 were calculated with *ProSMART* (Nicholls *et al.*, 2012 ▶) from the available PDB files after modification with *mrtailor* (Gruene, 2013 ▶). *BUSTER* (Bricogne *et al.*, 2011 ▶) was used with default settings.

## 3. Results and discussion   

### 3.1. Purification and crystallization   

The purification of complete, endogenous RNA polymerase I from *S. cerevisiae* was performed using a strain with a TAP tag on subunit AC40, which is shared between Pol I and Pol III. Accordingly, the two enzymes were isolated in the purification protocol with final yields of about 6 and 2 mg, respectively, from ∼1 kg wet weight. About 65 kg of yeast was processed to successfully complete the project.

**Figure 1:**
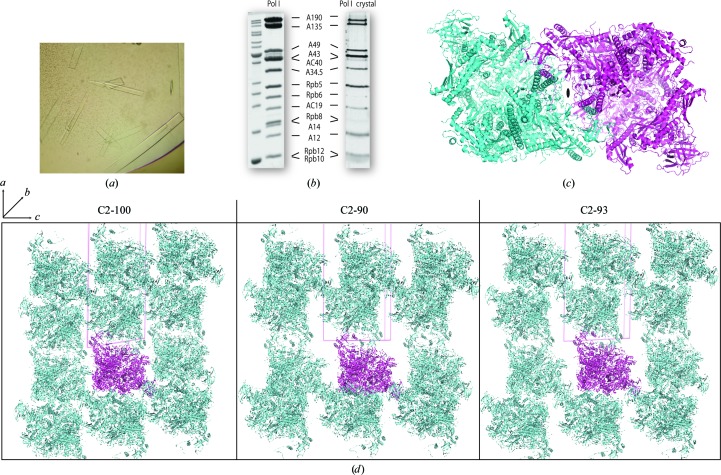
Crystallization of Pol I. (*a*) Typical Pol I crystals in space group *C*2 grown in sitting drops, as imaged 5 d after experimental setup. (*b*) 15% SDS–PAGE analysis of purified yeast Pol I (Coomassie staining) and a Pol I crystal (silver staining). The different relative intensity of the bands is owing to the different staining methods. Thicker bands of the MW marker (left lane) correspond to 50 and 10 kDa. (*c*) Pol I dimer formed by insertion of the A43 C-terminal tail of one monomer (pink) into the upper cleft of the neighbouring monomer (cyan) and *vice versa*. (*d*) Crystal packing in the different crystal forms as viewed from the *b* axis.

While crystallization trials with Pol III were unsuccessful, probably owing to substoichiometry of some subunits as observed by native mass spectrometry (Lane *et al.*, 2011 ▶), Pol I yielded hits in the very first sparse-matrix screenings. Successful precipitants ranged from alcohols (ethanol and butanediol) to ethylene glycol and different kinds of polyethylene glycol, but only crystals grown in ethylene glycol diffracted beyond 20 Å resolution. Optimization of the initial condition using standard grid screenings (pH *versus* ethylene glycol concentration) eventually yielded crystals that diffracted to 4 Å resolution (Fig. 1 ▶ *a*; Table 2 ▶). These crystals, hereafter named C2-93 according to their β angle, belonged to space group *C*2 and contained all 14 subunits as shown by SDS–PAGE (Fig. 1 ▶ *b*). New sparse-matrix screens aimed at finding alternative crystal forms with improved diffraction produced crystals in a condition with methylpentanediol (MPD). After grid-screen optimization, two additional crystal forms also belonging to space group *C*2, hereafter named C2-90 and C2-100 (Tables 3 ▶ and 4 ▶), were obtained from this condition. Crystals diffracting to around 3.5 Å resolution could be reproducibly grown in the three forms, but only one C2-100 crystal diffracted to 3.0 Å resolution. During the project, more than 2000 crystals were tested to collect successful data sets.

All crystal forms contain one molecule of the Pol I enzyme in the asymmetric unit. However, the crystallizing entity is a compact Pol I dimer (Fig. 1 ▶ *c*), as also observed in the simultaneously determined Pol I structure (Engel *et al.*, 2013 ▶) and in solution under defined conditions (Milkereit *et al.*, 1997 ▶). The twofold axis relating the monomers is coincident with the crystallographic *b* axis, which is the dimension that changes the least among the crystal forms. Reduction of *a* and *c* improves crystal packing, with the tightest packing occurring in C2-100, where both axes are reduced (Fig. 1 ▶ *d*). While compaction along *c* correlates with better diffraction power, it is also accompanied by partial disordering of the DNA-mimicking loop involved in transcriptional regulation (Fernández-Tornero *et al.*, 2010 ▶). Moreover, the C-terminal domain of subunit A12.2 involved in RNA cleavage is best ordered in the most loosely packed C2-93 crystal form.

### 3.2. Data collection and processing   

Native data sets were collected using methods for large unit cells (Mueller *et al.*, 2007 ▶) such as fine ϕ slicing to avoid spot overlapping. Also, as the crystals were radiation-sensitive, we employed minimal exposure times and took advantage of attenuators. When high resolution or high redundancy was required it was sometimes necessary to merge partially overlapping ϕ slices from different areas of the same crystal. This was possible because of the large size of our crystals (typically 0.7 × 0.3 × 0.05 mm) in comparison to the beam size (0.01–0.1 mm in diameter). In such cases, the correlation coefficient between data sets and largely deviating cell dimensions was used to remove outliers (Supplementary Table S1^1^). During the project, the PILATUS hybrid pixel detector was slowly introduced at different synchrotrons. This detector was critical in measuring high-resolution diffraction spots accurately, mainly owing to enhanced sensitivity and fine slicing (Mueller *et al.*, 2012 ▶).

Various approaches were used to extract maximal information for structure solution from the derivative data sets. In most cases, we collected inverse-beam data sets to minimize the adverse effects of radiation damage (Hendrickson *et al.*, 1989 ▶), especially for data sets collected at the peak energy of the fluorescence spectrum. However, for the critical ytterbium MAD experiment that yielded good-quality experimental phases, a different approach was used. Ytterbium has a strong white line whose signal drops beyond the absorption edge, making it possible to easily identify two inflection points (Supplementary Fig. S1). In our data-collection strategy, we first aligned the crystal along a twofold symmetry axis in order to collect Bijvoet pairs on the same image. This was allowed by the kappa-based goniometer available at the PROXIMA1 beamline at SOLEIL, after calculation of goniometer angles using the *XOalign* program (Legrand, 2009 ▶). Secondly, we collected one data set at each of the inflection points to properly measure the dispersive signal, which was the key to success in our phasing experiment. Finally, we collected a data set at the *L*_III_ absorption edge to gather anomalous information, followed by a reference data set at high energy from a different spot on the crystal.

The criterion to cut the resolution of a data set where 〈*I*/σ(*I*)〉 = 2 is arguably conservative and is intended not to include noise in the data. Noise would have a negative effect on the map quality and would hamper model building and refinement. The integration of noise can also affect the overall data-set quality because of the profile fitting performed by most modern data-processing software. As recently discussed, the resolution cutoff is by no means uniquely determined (Evans & Murshudov, 2013 ▶; Karplus & Diederichs, 2012 ▶). Once our model structures achieved decent quality with *R*_work_ and *R*_free_ values below 30%, we reprocessed our data to include all reflections down to where CC_1/2_ = 30%. This is the same limit as suggested for the cutoff of the anomalous signal used in experimental phasing strategies, where the inclusion of noise is a major obstacle to success (Schneider & Sheldrick, 2002 ▶). We confirmed by visual inspection of the electron-density maps that their quality improved, despite the high *R*_meas_ and low 〈*I*/σ(*I*)〉 values at this level (Tables 2 ▶, 3 ▶ and 4 ▶). The resolution difference between the 〈*I*/σ(*I*)〉 and the CC_1/2_ criterion is 0.35, 0.36 and 0.24 Å for C2-90, C2-93 and C2-100, respectively, which is close to recently suggested values (Luo *et al.*, 2014 ▶). Data statistics such as *R*_meas_ and 〈*I*/σ(*I*)〉 take all data into account, while refinement programs weight reflections in a more sophisticated manner, so that the contribution to noise is weighted down while real signal is kept to improve the map quality. We suggest that crystallographic tables list both the values at 〈*I*/σ(*I*)〉 = 2.0 and at the resolution limit used for refinement.

### 3.3. Initial 12-subunit model   

**Figure 2:**
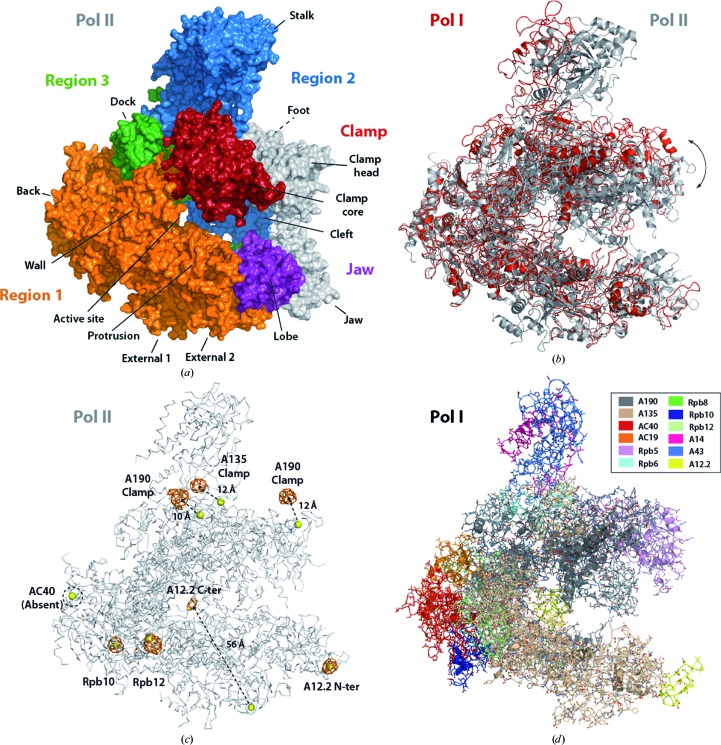
Molecular replacement leads to an initial model of Pol I. (*a*) Surface representation of the Pol II model (PDB entry 1wcm) showing the five different regions used in the sequential molecular replacement, with non-included domains in grey. Structural domains are labelled. (*b*) Superposition of the initial Pol I model onto the Pol II structure taking Region 1 as a reference. The wide conformation of the Pol I cleft is clearly observed. (*c*) Anomalous difference Fourier map at 8 Å resolution, contoured at 4σ, showing the seven Zn positions in Pol I, two of which helped in the localization of A12.2. The Pol II model is shown as a grey ribbon. (*d*) Initial 12-subunit model of Pol I obtained by molecular replacement followed by homology modelling. Subunit colours are as shown in the inset.

Starting phases were obtained by molecular replacement (MR) using a 4.0 Å resolution data set from a C2-93 crystal and the available atomic structures of Pol II. Initially, atomic structures of Pol II with 12 and ten subunits were used, but both yielded negative log-likelihood gain (LLG) values. Since we expected conformational differences between Pol I and Pol II, the Pol II model (PDB entry 1wcm; Armache *et al.*, 2005 ▶) was divided into five regions similar to the modules described in Cramer *et al.* (2001 ▶) (see §[Sec sec2]2 and Fig. 2 ▶ *a*) and subjected to sequential molecular replacement. ‘Region 1’ was found first, with a *Z*-score of 8.9 and an LLG value of 16. The small LLG value reflects the fact that only C^α^ atoms were employed during sequential MR. Placement of ‘Region 2’, comprising the shelf and stalk modules, increased the *Z*-score and LLG values to 14.0 and 42, respectively. The lower *Z*-score obtained when the shelf was used alone suggested that the position of the stalk was roughly correct. Positioning of ‘Region 3’ slightly lowered the *Z*-score to 13.7 but significantly increased the LLG to 69, indicating correct location of this domain, as confirmed by the biological consistency of the solution. Moreover, when this solution containing the three regions was subjected to simple MR, the overall *Z*-score and LLG values were 19.0 and 70, respectively. In contrast, subsequent molecular replacement with the clamp core or lobe modules did not yield meaningful solutions.

Using the three-region MR solution as a template, we built a truncated version of the Pol I structure comprising 11 subunits (Table 1 ▶, first 11 rows). The five subunits that are common to the three eukaryotic RNA polymerases, Rpb5, Rpb6, Rpb8, Rpb10 and Rpb12, were maintained. The available crystal structure of the Pol I stalk (Kuhn *et al.*, 2007 ▶), comprising subunits A43 and A14, was placed by superposition of A43 onto Pol II subunit Rpb7. For A190, A135, AC40 and AC19, homology modelling of conserved domains was employed. The resulting model showed a wide conformation of the DNA-binding cleft. When the crystal structure of Pol II was superposed taking ‘Region 1’ as a reference, the remaining half of the enzymes no longer superposed (Fig. 2 ▶ *b*), explaining why MR with the entire Pol II model was not successful. Importantly, this unique conformation of the enzyme is one of the main characteristics of dimeric Pol I (Fernández-Tornero *et al.*, 2013 ▶), demonstrating that useful biological information can be extracted through X-ray crystallographic experiments even in the absence of a refined atomic model.

At this stage, three entire subunits were missing in our model: A12.2, A49 and A34.5. An atomic model of the A49/A34.5 dimerization module was built from the available crystal structure of human TFIIF subunits Rap74/Rap30 (Gaiser *et al.*, 2000 ▶) using *MODELLER* (Eswar *et al.*, 2006 ▶). Nevertheless, all MR trials were unsuccessful. Subunit A12.2 is made by two Zn ribbons, each homologous to the N-terminal domain of Pol II subunit Rpb9 and the C-terminal domain of TFIIS, connected by an extended linker (Fernández-Tornero *et al.*, 2013 ▶). Therefore, in order to locate A12.2 in our structure, we collected a data set at the *K* absorption edge of Zn using a C2-93 native crystal (Table 2 ▶) and calculated Zn anomalous maps by cross-difference Fourier analysis using the model phases as a reference. Seven Zn positions with σ values above 4 were identified, correlating with the expected Pol I composition (Fig. 2 ▶ *c*). Two Zn^2+^ ions belong to subunits Rpb10 and Rpb12 shared by Pol I and Pol II. Three other Zn^2+^ ions in the clamp are conserved between these enzymes but are shifted in Pol I by about 10 Å from the expected position, further confirming the wide conformation of the DNA-binding cleft. The two remaining Zn^2+^ ions belong to the A12.2 Zn ribbons and mark their positions. Homology modelling using the N-terminal domain of Rpb9 and the C-terminal domain of TFIIS allowed us to obtain an initial 12-subunit model of Pol I comprising 59% of the enzyme residues (Fig. 2 ▶ *d*).

### 3.4. Experimental phasing and density modification   

**Figure 3:**
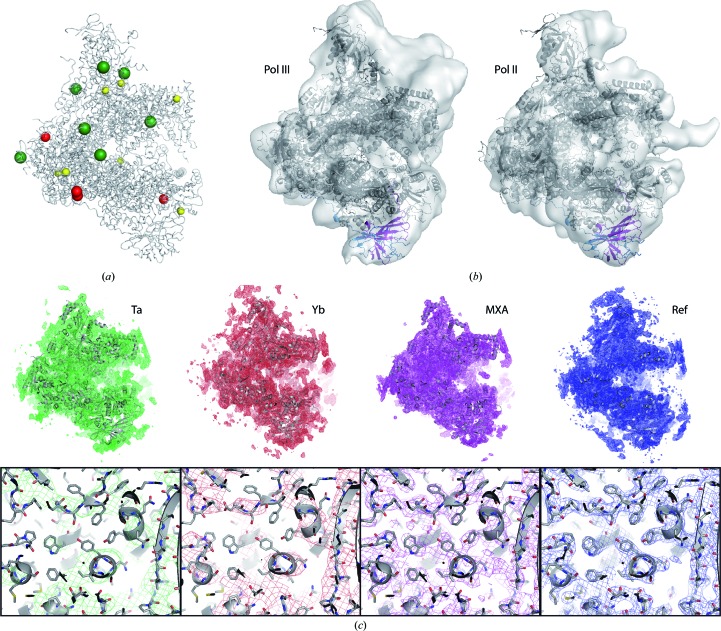
Experimental phasing and density modification. (*a*) Binding positions of the different derivatives identified by cross-difference Fourier using available model phases. Tantalum, ytterbium and zinc are shown in green, red and yellow, respectively. (*b*) Pol I model fitted into the EM reconstructions of Pol III (EMD-1802) and the Pol II pre-initiation complex (EMD-2306), showing comparable positions for the A49/A34.5 heterodimer (violet/blue) and its Pol III and Pol II counterparts C37/C53 and TFIIF, respectively. (*c*) General view (top) and zoom (bottom) of the electron-density maps (contoured at 1σ) obtained during the Pol I structure determination. From left to right: solvent-flattened map after tantalum phasing in C2-93 (Ta), solvent-flattened map after ytterbium phasing in C2-90 (Yb), map resulting from a multi-crystal averaging in C2-93 (MXA) and final refined map in C2-100 (Ref). While the initial Ta map showed the position of the A49/A34.5 dimerization module, the Yb map showed improved density for the main chain and the MXA map showed details of side chains.

Refinement of the initial 12-subunit model was hampered by the poor quality of the phases. In order to obtain experimental phase information, we first collected a data set at the Ta absorption edge from a Ta_6_Br_12_ derivative belonging to crystal form C2-93 that diffracted to 6.65 Å resolution (Table 2 ▶). Using the available model phases, we found seven Ta_6_Br_12_ positions with σ values above 6 (highest peak = 15.4σ), mostly located at charged surface regions (Fig. 3 ▶ *a*, green spheres). Attempts to determine the phases using this data set alone failed, but SIRAS in combination with the 4.0 Å resolution native data provided acceptable phases at low resolution, which were extended to the resolution of the native data through solvent flattening. While the map quality was still moderate, as shown by discontinuous density for β-strands, a large piece of additional density was readily visible next to the lobe element (Fig. 3 ▶ *c*, green map). We manually fitted the homology model of the A49/A34.5 dimerization module (see above) into this density, assisted by cross-linking results between this module and the lobe (Jennebach *et al.*, 2012 ▶). Interestingly, the position of this module agrees nicely with that of the equivalent Pol III heterodimer (Fernández-Tornero *et al.*, 2010 ▶) and also TFIIF bound to Pol II (He *et al.*, 2013 ▶), as observed by electron cryomicroscopy (Fig. 3 ▶ *b*). This result confirmed the hypothesis that the three nuclear RNA polymerases share a TFIIF-like dimerization module, further showing how relevant biological information can be extracted from low-resolution crystallographic experiments. Interestingly, a similar module has been found in TFIIIC, a Pol III-specific transcription factor (Taylor *et al.*, 2013 ▶).

To improve the experimental phases, we tested several lanthanoid complexes (Talon *et al.*, 2011 ▶), finding success in the case of a C2-90 crystal soaked with Yb-HPDO3A. A multi-wavelength anomalous dispersion (MAD) data set was obtained at wavelengths corresponding to the peak, the rising and falling inflection points of the Yb *L*_III_ absorption edge and a high-energy remote position (Table 3 ▶). Using the available model phases, we identified four Yb positions with σ values above 6 (highest peak = 20.1σ) located on charged surface regions (Fig. 3 ▶ *a*, red). Interestingly, two of the sites form a dimer, as previously observed for this kind of compound (Girard *et al.*, 2002 ▶). SAD, SIRAS and MAD phasing using different wavelength combinations were tested to generate phases at 4.1 Å resolution, followed by solvent flattening. The best results were obtained with MAD using the peak, rising and falling inflection and high-energy remote wavelengths. The resulting map greatly improved the definition of the main chain, especially in regions containing β-strands or loops (Fig. 3 ▶ *c*, red map).

A two-step strategy was used to enhance the map quality and further remove model bias. Firstly, we used the prime-and-switch protocol, which maximizes the map likelihood using an unbiased probability estimate (Terwilliger, 2004 ▶) and can be combined with *B*-factor sharpening. We found it useful to analyse maps with different *B*-factor sharpening to examine high-resolution and low-resolution features. The resulting maps proved helpful in discovering wrongly traced main-chain regions as well as in rotamer selection. Secondly, to overcome the lack of NCS, we deployed multi-crystal averaging using the model and experimental phases in C2-90 and C2-93. While the phases in C2-90 were of excellent quality at this stage, further optimization was required in the case of C2-93. New model phases in this crystal form were obtained by rigid-body refinement of the C2-90 model after division into 28 groups (see §[Sec sec2]2). The resulting model phases were then combined with experimental information in an improved MIRAS protocol that included a new native data set to 3.6 Å resolution, the original tantalum derivative, a new ytterbium derivative collected at the *L*_III_ peak and a data set collected at the absorption peak of Zn (Table 2 ▶). Multi-crystal averaging using prime-and-switch maps in C2-93 and C2-90 for starting coefficients yielded combined maps of excellent quality (Fig. 3 ▶ *b*, purple map), with well defined density for most side chains and certain main-chain areas where previous density was poor.

### 3.5. Model building and refinement   

The initial model was less than 60% complete and had strong model bias towards the Pol II structure used for molecular replacement. Moreover, serious tracing errors were present owing to the limited map quality. While refinement with the default settings of the programs generally lowered the *R*_work_ and *R*_free_ values, the resulting model showed poor geometry and difference maps did not allow major extensions or corrections. To overcome this situation, several parameters were tuned with *REFMAC*5 (Nicholls *et al.*, 2012 ▶) and *PHENIX* (Adams *et al.*, 2010 ▶), including the geometry weighting, the total number of cycles, the use of experimental phase information, the bulk-solvent estimation and the application of external restraints for secondary-structure elements. The best results were obtained with a high number of cycles (up to 100–500 cycles in *REFMAC*5 and 15 macrocycles in *PHENIX*), strong geometry weights and the use of experimental phase information (Pannu *et al.*, 1998 ▶) and external geometric restraints (Murshudov *et al.*, 2011 ▶). To reduce the model bias from external restraints, nonconserved regions were removed from the reference Pol II structure using the program *mrtailor* (Gruene, 2013 ▶). Typically, about 15 different settings were tested and rated based on the stereochemical quality of the model and the visual quality of the maps.

**Figure 4:**
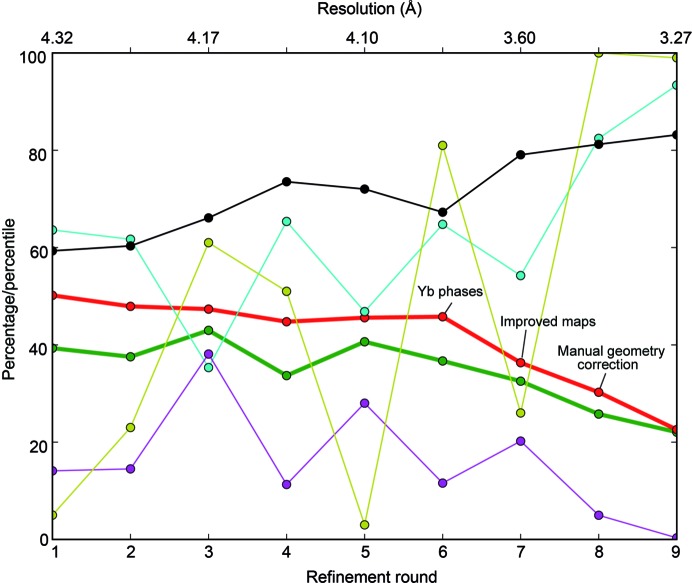
Evolution of structural refinement in C2-93. *R*_work_ (green), *R*_free_ (red), Ramachandran favoured residues (blue), Ramachandran outliers (purple), *MolProbity* percentile (yellow) and residues built (black), all expressed in percentages, are plotted per refinement cycle. Rounds 1–6 were run against native 1 at increasing resolution cutoffs, while rounds 7–­9 were run against native 2 using the same strategy (Table 2 ▶).

The initial advances were slow and mostly concerned model completion (Fig. 4 ▶, rounds 1–6), mainly owing to the low resolution of the experimental phase information and the low quality and bias of the 2*mF*_o_ − *DF*_c_ maps. Therefore, model building at this stage was conservative to avoid mistakes such as register shifts, incorrect tracing or wrong chain assignment. Atomic models of homologous proteins were displayed to assist building, such as the structures of *S. cerevisiae* Pol II (Armache *et al.*, 2005 ▶), *Sulfolobus shibatae* Pol (Wojtas *et al.*, 2012 ▶), the *Candida glabrata* A49/A34.5 dimerization module (Geiger *et al.*, 2010 ▶) and the *S. cerevisiae* A43/A14 stalk sub­complex (Kuhn *et al.*, 2007 ▶). For conserved domains, a similar tracing coherent with the experimental map was used. In nonconserved regions, polyalanine stretches were built and the sequence was only assigned if the presence of predicted secondary-structure elements and bulky side chains supported the register with high confidence.

Once we had obtained higher resolution experimental phases and produced multi-crystal averaging maps, progress was faster and the model steadily improved (Fig. 4 ▶, rounds 7–8). Artefacts from building such as *cis*-peptides were removed, while rotamer outliers were corrected, preferring those similar to the reference structures when the maps were unclear. In the final rounds, the need for phase improvement over the high-quality model phases was obviated, while occasionally remaining errors were overcome using maps other than those arising from refinement, *i.e.* prime-and-switch and multi-crystal averaging. During the entire procedure, we progressively improved the resolution of our crystals, which was critical to obtain maps of better quality and thus more accurate atomic models. The best-diffracting crystal, with a resolution of 3 Å and belonging to C2-100 (Table 4 ▶), was used for the remaining geometry corrections. Final refinement with *BUSTER* (Fig. 3 ▶ *c*, blue map) yielded improved *R* values and models of excellent quality in all three crystal forms, as shown by *MolProbity* (Chen *et al.*, 2010 ▶).

### 3.6. Sequence markers for trace confirmation and active-site location   

**Figure 5:**
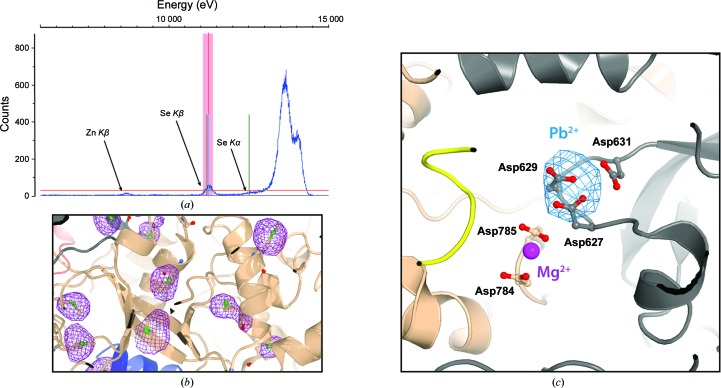
Use of sequence markers. (*a*) Excitation scan at the Se *K* edge from a SeMet-labelled Pol I crystal indicating the presence of Se in the sample. Peaks corresponding to Zn *K*β, Se *K*β and Se *K*α are also labelled. (*b*) The anomalous difference map localizing Se peaks is shown in violet. (*c*) Anomalous difference map (blue) from a lead-derivative crystal showing the location of the Pol I active site. The primary Mg^2+^ ion is coordinated by a triad of Asp residues in subunit A190. The putative position of the secondary Mg^2+^ ion is shown in purple next to the Asp residues in subunit A135.

To solve the remaining ambiguities and fully confirm our tracing, we performed a series of anomalous data-collection experiments on atoms that mark the positions of specific amino acids. Firstly, we undertook the challenge of using native crystals to locate the position of S atoms from cysteines and methionines in the structure. Owing to the weak scattering power of S atoms, we aimed to enrich the anomalous signal by harvesting an enormous amount of data at 1.77 Å resolution, where the anomalous scattering power of S is about 0.7 e^−^. The best data set was collected from a C2-100 crystal (Table 4), allowing the location of 77 of 174 modelled S atoms (44%) within a distance of 3.0 Å from methionine or cysteine residues (Supplementary Table S2). This moderate success led us to labelling experiments using selenomethionine (SeMet). We produced and purified partially labelled SeMet Pol I, which yielded crystals in the same conditions as the native protein, although they took 3–4 days longer to grow (Fig. 5 ▶ *a*). A highly redundant data set was collected from a C2-90 crystal at the Se *K* edge (Table 3 ▶). This procedure allowed the location of 90 of 102 modelled Se atoms (88%) within a distance of less than 2.3 Å from methionine residues (Supplementary Table S3). This result was very useful to confirm the main-chain tracing (Fig. 5 ▶ *b*), especially in poorly defined regions in the complex, such as the A49/A34.5 dimerization module.

Following a similar strategy, we also aimed to identify the active site of the enzyme. In Pol II, a Mg^2+^ ion coordinated by three aspartate residues in subunit Rpb1 is directly involved in catalysis, while a secondary Mg^2+^ ion has been proposed to participate in NTP substrate binding (Brueckner *et al.*, 2009 ▶; Vassylyev *et al.*, 2007 ▶). Our electron-density maps only showed metal-like density in crystal form C2-100, where it was possible to model an Mg^2+^ ion next to two aspartate residues in subunit A135 (Fig. 5 ▶ *c*, purple sphere), which may correspond to the secondary metal in Pol II. In order to confirm that the primary metal-binding site was conserved in Pol I, we prepared a lead derivative because Pb^2+^ is coordinated with similar geometry to Mg^2+^ (Holloway & Melnik, 1997 ▶). Anomalous maps calculated from a data set collected at the lead absorption edge demonstrate that the active-site residues (Asp627, Asp629 and Asp631 in subunit A190) are indeed capable of coordinating Mg^2+^ (Fig. 5 ▶ *c*). Therefore, a catalytic mechanism involving two metals, equivalent to that proposed for Pol II and bacterial Pol (Brueckner *et al.*, 2009 ▶; Vassylyev *et al.*, 2007 ▶), appears to be likely.

### 3.7. Proposed workflow   

The final models show excellent statistics, with *MolProbity* scores in the 100th percentile (C2-90), 98th percentile (C2-93) and 99th percentile (C2-100), *i.e.* among the best structures at comparable resolution. Two reasons may explain these results. Firstly, the inclusion of weak but significant data improves the model quality, as suggested previously (Evans & Murshudov, 2013 ▶; Karplus & Diederichs, 2012 ▶). Secondly, careful model building taking into account different electron-density maps, conserved regions of homologous proteins, sequence markers and stereochemistry allows the construction of good models even at limited resolution with poor initial phase information.

**Figure 6:**
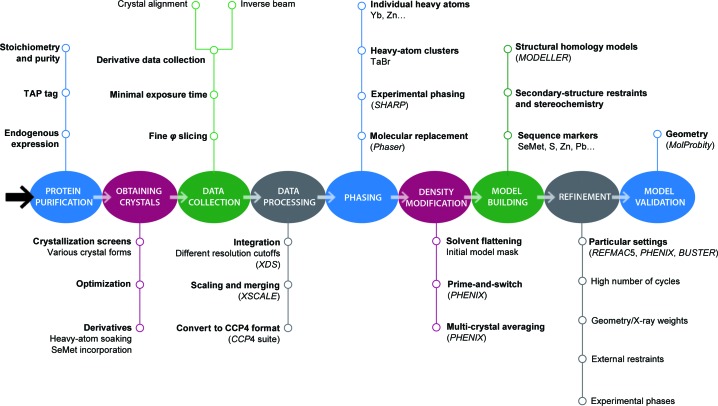
Workflow for PolI structre determination. The different stages, main steps and possible alternatives in decision making are shown.

Despite the risk of over-interpretation when dealing with atomic models built from low-resolution diffraction images, the authors believe that efforts can and should be taken to obtain critical biological information from the available data. With this idea in mind, we propose a general workflow that integrates the different strategies used during the current project (Fig. 6 ▶). Alternative experimental approaches may also be considered depending on the complex under study. For example, when recombinant production is possible, systematic SeMet labelling of specific residues can assist in chain tracing (Oubridge *et al.*, 2009 ▶). Distance restraints such as those obtained from cross-linking coupled to mass spectrometry (Rappsilber, 2011 ▶) can be useful during the initial steps of model building. Finally, available EM reconstructions may prove useful at different stages of the X-ray structure-determination process. Successful examples of such combined approaches include the use of EM maps for molecular replacement, heavy-atom location and phase extension (Ban *et al.*, 1998 ▶; Xiong, 2008 ▶). We hope that our work will inspire other scientists that endeavour the difficult task of analysing large macromolecular assemblies through X-ray crystallography.

## Supplementary Material

Supporting Information.. DOI: 10.1107/S1399004714015788/tz5059sup1.pdf

## Figures and Tables

**Table 1:** Subunit composition of yeast RNA polymerases

Pol II	Pol I	Pol III	Chain ID
Rpb1	A190	C160	*A*
Rpb2	A135	C128	*B*
Rpb3	AC40†	AC40†	*C*
Rpb11	AC19†	AC19†	*K*
Rpb5 (ABC27)‡	Rpb5 (ABC27)‡	Rpb5 (ABC27)‡	*E*
Rpb6 (ABC23)‡	Rpb6 (ABC23)‡	Rpb6 (ABC23)‡	*F*
Rpb8 (ABC14.5)‡	Rpb8 (ABC14.5)‡	Rpb8 (ABC14.5)‡	*H*
Rpb10 (ABC10β)‡	Rpb10 (ABC10β)‡	Rpb10 (ABC10β)‡	*J*
Rpb12 (ABC10α)‡	Rpb12 (ABC10α)‡	Rpb12 (ABC10α)‡	*L*
Rpb4	A14	C17	*D*
Rpb7	A43	C25	*F*
Rpb9 + TFIIS§	A12.2	C11	*I*
TFIIFα§	A49-N	C37	*M*
TFIIFβ§	A34.5	C53	*N*
TFIIEα§	—	C82	—
TFIIEβ§	A49-C	C34	Disordered
—	—	C31	—

† Shared by Pol I and Pol III.

‡ Shared by Pol I, Pol II and Pol III.

§ TFIIS, TFIIF and TFIIE are not Pol II subunits but transcriptions factors that bind transiently.

**Table 2:** Data-collection, processing and phasing statistics for crystal form C2-93 Values in parentheses are for the outer shell.

			Native 3 (Zn)	Ta_6_Br_12_	HDPOA-Yb	Phenyl lead
	Native 1 (MR and SIRAS)	Native 2 (MIRAS)	Zn peak	Ta peak	Yb *L*_III_ peak	Pb peak
Data collection						
Space group	*C*2	*C*2	*C*2	*C*2	*C*2	*C*2
Unit-cell parameters						
*a* (Å)	427.4	425.2	426.8	425.1	425.6	423.1
*b* (Å)	141.6	140.6	141.0	141.0	141.2	141.2
*c* (Å)	142.3	139.7	141.3	140.6	140.3	140.2
β (°)	93.93	93.35	94.42	94.43	93.50	93.73
Beamline	ID23-2, ESRF	PX1, SOLEIL	ID23-1, ESRF	ID14-4, ESRF	PX1, SOLEIL	ID29, ESRF
Detector	MAR CCD 225	PILATUS 6M	ADSC Q315r	ADSC Q315r	PILATUS 6M	ADSC Q315r
Wavelength (Å)	0.87260	1.07160	1.28270	1.25452	1.38530	0.94770
*E* (eV)	14208	11570	9666	9883	8950	13083
ϕ_total_ (°)	140	580	360	360	360	360
Δϕ (°)	0.5	0.1	0.5	1.0	0.2	1.0
Data processing						
Resolution (Å)	50.0–4.00 (4.25–4.00)	97.3–3.27 (3.35–3.27)	50.0–4.20 (4.43–4.20)	58.9–6.65 (6.83–6.65)	60.0–4.70 (4.90–4.70)	50.0–5.75 (5.90–5.75)
Total reflections	224284 (37699)	1393070 (105561)	460573 (68543)	114182 (7769)	282648 (21513)	173563 (11867)
Unique reflections†	71035 (11822)	127066 (9384)	120171 (18270)	29702 (2104)	81899 (6103)	45593 (3417)
Multiplicity	3.1 (3.2)	11.0 (11.2)	3.8 (3.8)	3.8 (3.7)	3.5 (3.5)	3.8 (3.5)
Completeness (%)	98.9 (99.5)	100.0 (99.9)	98.7 (93.2)	99.3 (97.0)	99.5 (99.5)	99.2 (99.7)
*R*_meas_ (%)	20.0 (113.5)	16.2 (583.8)	22.7 (172.3)	15.1 (178.9)	12.6 (134.9)	16.6 (136.4)
〈*I*/σ(*I*)〉	6.04 (1.27)	10.85 (0.51)	5.26 (1.16)	10.02 (1.00)	9.13 (1.35)	8.63 (1.36)
CC_1/2_	0.996 (0.372)	0.999 (0.317)	0.992 (0.416)	0.997 (0.265)	0.997 (0.580)	0.995 (0.425)
Wilson *B* (Å^2^)	127.6	122.3	159.5	391.5	232.2	293.6
Phasing/markers						
Sites	N/A	N/A	7 Zn	7 Ta + 7 Zn	4 Yb	1 Pb
SIRAS FOM (exp/DM)	0.08/0.64					N/A
MIRAS FOM (exp/DM)		0.21/0.67				N/A

† For anomalous data, Friedel pairs were treated as separate reflections.

**Table 3:** Data-collection, processing and phasing statistics for crystal form C2-90 Values in parentheses are for the outer shell.

		SeMet	HDPOA-Yb			
	Native 4	Se peak	Yb *L*_III_ peak	*L*_III_ rising inflection	*L*_III_ falling inflection	Remote
Data collection						
Space group	*C*2	*C*2	*C*2			
Unit-cell parameters						
*a* (Å)	401.3	403.5	404.5			
*b* (Å)	139.9	140.4	140.4			
*c* (Å)	141.0	142.1	143.0			
β (°)	90.41	90.03	90.33			
Beamline	PX1, SOLEIL	PX1, SOLEIL	PX1, SOLEIL			
Detector	PILATUS 6M	PILATUS 6M	PILATUS 6M			
Wavelength (Å)	0.98011	0.97903	1.38545	1.38592	1.38499	0.95372
*E* (eV)	12650	12664	8949	8946	8952	13000
ϕ_total_ (°)	400	1520	200	180	180	360
Δϕ (°)	0.1	0.1	0.1	0.1	0.1	0.1
Data processing						
Resolution (Å)	79.9–3.36 (3.44–3.36)	71.1–3.59 (3.69–3.59)	49.2–4.00 (4.24–4.00)	49.2–4.00 (4.24–4.00)	49.2–4.00 (4.24–4.00)	49.2–4.00 (4.10–4.00)
Total No. of reflections	846467 (63472)	2592789 (126603)	258956 (40241)	231002 (36126)	231045 (36026)	466906 (34716)
Unique reflections†	110892 (8124)	181271 (12905)	130172 (20513)	128213 (20343)	128253 (20291)	132998 (9862)
Multiplicity	7.6 (7.8)	14.3 (9.8)	2.0 (2.0)	1.8 (1.8)	1.8 (1.8)	3.5 (3.5)
Completeness (%)	99.8 (97.6)	99.6 (95.2)	97.2 (95.2)	96.4 (95.2)	96.3 (94.9)	99.7 (99.7)
*R*_meas_ (%)	21.3 (254.7)	19.9 (391.7)	10.7 (135.4)	9.2 (90.2)	9.7 (95.6)	12.9 (190.2)
〈*I*/σ(*I*)〉	8.52 (0.83)	11.20 (0.93)	7.08 (0.94)	8.05 (1.36)	7.80 (1.29)	8.01 (0.92)
CC_1/2_	0.996 (0.303)	0.999 (0.375)	0.997 (0.489)	0.998 (0.645)	0.997 (0.624)	0.997 (0.435)
Wilson *B* (Å^2^)	97.2	159.3	160.8	151.7	150.8	159.4
Phasing/markers						
Sites	N/A	90 Se	4 Yb	4 Yb	4 Yb	4 Yb + 7 Zn
FOM (exp/DM)	N/A	N/A	0.27/0.68			

† For anomalous data, Friedel pairs were treated as separate reflections.

**Table 4:** Data-collection and processing statistics for crystal form C2-100

	Native 5	Native 6 (S-SAD)
Data collection		
Space group	*C*2	*C*2
Unit-cell parameters		
*a* (Å)	400.4	405.1
*b* (Å)	140.2	141.1
*c* (Å)	122.9	123.8
β (°)	100.14	100.15
Beamline	P14, DESY PETRA III	PX1, SOLEIL
Detector	PILATUS 6M	PILATUS 6M
Wavelength (Å)	0.97626	1.6531
*E* (eV)	12700	7500
ϕ_total_ (°)	360	2180
Δϕ (°)	0.1	0.1
Data processing		
Resolution (Å)	77.6–3.03 (3.11–3.03)	70.0–3.50 (3.59–3.50)
Total reflections	895115 (63225)	3527564 (230140)
Unique reflections†	128299 (9259)	170279 (12574)
Multiplicity	7.0 (6.8)	20.7 (18.3)
Completeness (%)	98.5 (96.3)	99.9 (99.9)
*R*_meas_ (%)	11.7 (240.2)	22.6 (428.4)
〈*I*/σ(*I*)〉	13.20 (0.86)	12.67 (0.97)
CC_1/2_	0.999 (0.307)	0.999 (0.455)
Wilson *B* (Å^2^)	96.0	120.2
Phasing/markers		
Sites	N/A	77

† For anomalous data, Friedel pairs were treated as separate reflections.

## Footnotes

1 Supporting information has been deposited in the IUCr electronic archive (Reference: TZ5059).
