# Supplementary material for: Solving the RNA polymerase I structural puzzle
Source: Acta Crystallogr D Biol Crystallogr. 2014 Sep 27;70(Pt 10):2570–82. doi: 10.1107/S1399004714015788 (PMC4188003; doi:10.1107/S1399004714015788)
Supplement: Supplementary file 1 [file d-70-02570-sup1.pdf]

Acta Crystallographica Section D

Volume 70 (2014)

Supporting information for article:

Solving the RNA polymerase I structural puzzle

María Moreno-Morcillo, Nicholas M. I. Taylor, Tim Gruene, Pierre Legrand, Umar J. Rashid, Federico M. Ruiz, Ulrich Steuerwald, Christoph W. Müller and Carlos Fernández-Tornero

Table S1 Example of data set merging

Datasets[1] i	j	Common reflections	Correlation (i,j)	Common intensity ratio (i/j)	B-factor (i,j)
4	5	4846	0.88	0.90	0.30
5	6	227	0.92	0.84	-0.01
1	5	4635	0.92	0.84	0.21
3	5	1445	0.94	1.08	0.11
4	6	858	0.96	0.92	0.21
2	5	181	0.97	1.12	0.01
1	6	8636	0.98	1.06	1.77
2	3	5999	0.98	0.94	1.01
3	4	7486	0.98	0.92	2.71
3	6	7987	0.98	1.07	-0.79
1	4	8630	0.98	0.93	-0.12
1	2	7805	0.99	1.07	1.16
2	4	719	0.99	1.08	-0.26
2	6	9007	0.99	1.01	-0.01
1	3	11219	0.99	1.03	-0.41

[1]In this example, dataset 5 was excluded

Table S2 Localization of S atoms with anomalous maps

Subunit	Residue (Met)	Closest atom	Peak intensity (σ)	Residue (Cys)	Closest atom	Peak intensity (σ)
**Rpa190**	471	SD	3.16	85	SG	3.10
	600	SD	3.22	333	SG	3.13
	601	SD	3.21	1255	SG	3.56
	977	SD	3.51	828	SG	3.62
	960	SD	3.54	999	SG	4.01
	944	SD	3.68	62	SG	4.11
	357	SD	3.78	1069	SG	4.39
	589	SD	3.84	922	SG	4.72
	925	SD	3.92	699	SG	4.67
	1	SD	3.93	102	SG	5.51
	623	SD	3.95	65	SG	5.56
	1170	SD	4.00	236	SG	5.95
	472	SD	4.26	233	SG	5.98
	1294	SD	4.28	105	SG	6.51
	1529	SD	4.38	72	SG	7.74
	1603	SD	4.49			
	635	SD	4.50			
	1613	SD	4.87			
**Rpa135**	373	SD	3.10	734	SG	3.03
	649	SD	3.34	170	SG	3.27
	1074	CB	3.38	584	SG	3.50
	275	SD	3.39	585	SG	3.90
	446	SD	3.41	1131	SG	6.46
	215	SD	3.44	1104	SG	6.93
	175	SD	3.51	1128	SG	7.18
	716	SD	3.66	1107	SG	7.34

	762	SD	3.72			
	377	SD	3.80			
	936	SD	3.83			
	958	SD	4.11			
	721	SD	4.18			
	461	SD	4.25			
	205	SD	4.60			
	1057	SD	4.72			
	672	SD	5.23			
**AC40**				250	SG	3.14
				140	SG	3.97
**ABC23**	122	SD	4.23			
**A43**	71	SD	3.64	118	SG	3.08
	62	SD	4.57			
	52	SD	4.78			
**ABC10α**				51	SG	5.34
				34	SG	6.30
				48	SG	8.18
**ABC10β**	49	SD	3.91	7	SG	5.24
				10	SG	5.80
				45	SG	6.18
				46	SG	8.57
**A12.2**				114	SG	3.65
				30	SG	3.66
				13	SG	4.67
				10	SG	5.12

Table S3 Localization of Se atoms with anomalous maps

Subunit	Residue (Met)	Closest atom	Peak intensity (σ)	Distance (Å)	Residue (Met)	Closest atom	Peak intensity (σ)	Distance (Å)
**Rpa190**	1	SD	5.60	0.9	977	SD	5.91	0.6
	191	-	-	-	1000	SD	3.65	0.5
	238	SD	5.00	1.0	1049	SD	5.50	0.7
	357	SD	7.72	0.1	1063	CG	3.96	0.9
	424	SD	5.28	1.7	1170	SD	6.79	0.4
	471	SD	7.94	0.4	1175	CE	4.16	0.3
	472	SD	6.02	0.3	1200	SD	6.75	1.0
	549	SD	5.44	0.9	1227	SD	5.40	1.0
	589	SD	4.68	0.9	1238	SD	5.63	0.2
	600	SD	5.43	1.1	1294	SD	3.88	0.3
	601	SD	6.85	0.5	1395	-	-	-
	633	SD	6.18	0.4	1439	SD	5.69	1.5
	635	SD	7.51	0.7	1485	SD	4.63	0.4
	744	SD	6.41	1.0	1529	SD	5.31	0.6
	830	SD	5.06	0.9	1588	CE	4.53	1.3
	917	SD	4.58	1.8	1589	SD	6.94	0.4
	925	SD	4.51	1.0	1603	-	-	-
	928	SD	5.83	0.4	1611	SD	4.77	1.1
	944	SD	5.30	0.7	1613	SD	6.49	0.6
	960	CE	6.84	0.8	-	-	-	-
**Rpa135**	108	SD	4.45	0.8	716	SD	7.04	1.4
	164	SD	5.43	0.4	721	SD	5.28	0.4
	175	CB	4.56	0.6	726	SD	6.07	0.7
	205	SD	5.85	0.4	762	SD	4.98	1.1
	215	SD	5.66	0.8	783	SD	5.67	1.1
	275	SD	5.31	1.4	787	CE	5.24	0.4

	373	SD	7.22	0.7	803	SD	6.32	0.8
	377	-	-	-	936	SD	5.96	0.3
	411	CE	4.65	1.3	958	SD	5.25	0.5
	430	SD	4.03	1	962	SD	5.81	0.4
	436	-	-	-	1013	SD	8.22	0.3
	446	-	-	-	1039	-	-	-
	451	-	-	-	1057	CE	4.47	0.9
	461	SD	5.79	0.4	1074	SD	6.39	0.9
	502	SD	5.26	1.3	1133	SD	6.06	0.6
	649	SD	7.47	0.4	1192	SD	4.52	0.9
	672	CE	4.79	0.8	-	-	-	-
**AC40**	71	SD	5.72	0.6	308	SD	6.26	0.3
	112	SD	4.42	0.9	-	-	-	-
**ABC27**	1	-	-	-	75	SD	7.29	1.2
	22	SD	6.41	0.5	93	SD	3.93	0.6
	50	-	-	-	121	CG	4.40	2.3
	57	SD	4.41	1.5	215	CE	4.35	1.5
	58	SD	5.42	0.9				
**ABC23**	85	SD	7.74	0.8	122	SD	4.79	0.9
	103	SD	6.95	1.0	-	-	-	-
**A43**	52	CE	3.82	1.2	71	SD	4.07	0.4
	62	SD	5.38	0.5	-	-	-	-
**ABC14.5**	97	SD	5.07	1.0	123	SD	6.35	1.0
**A12.2**	94	SD	6.81	0.4				
**ABC10α**	1	SD	5.60	0.4	49	SD	6.48	0.3
**AC19**	81	SD	4.08	0.5	142	CE	4.72	0.7
	125	SD	4.18	0.7	-	-	-	-
**A34.5**	80	SD	4.30	0.8	107	CE	4.28	1.0
	90	-	-	-	-	-	-	-

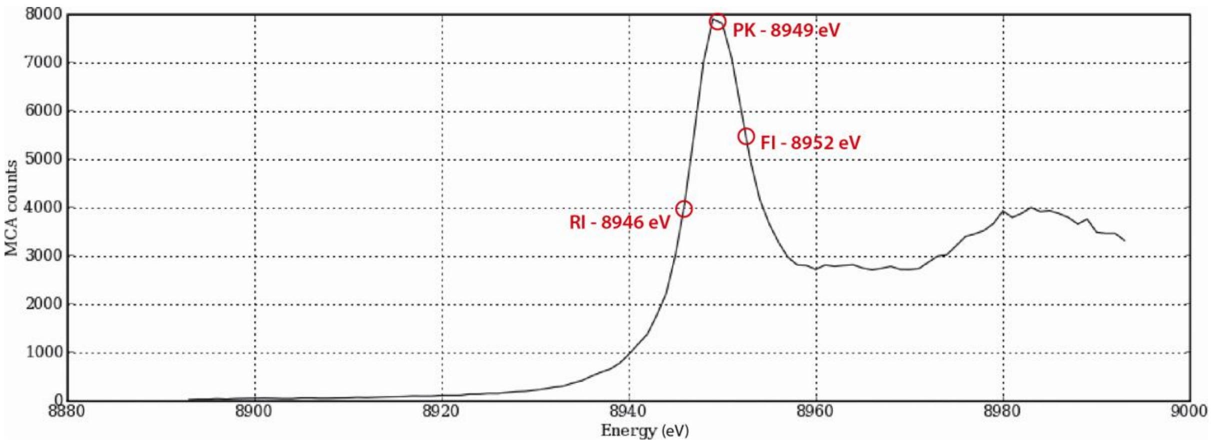

Figure S1 Data collection strategy for MAD experiments. Anomalous scattering scan of a Pol I crystal soaked for 2 min with Yb-HPDO3A and back-soaked in cryo-protectant solution. Wavelengths chosen for the MAD experiment are in red, with RI, PK and FI corresponding to the raising inflection, peak and falling inflection points, respectively.
